# System Dynamics to Model the Unintended Consequences of Denying Payment for Venous Thromboembolism after Total Knee Arthroplasty

**DOI:** 10.1371/journal.pone.0030578

**Published:** 2012-04-20

**Authors:** Mathias Worni, Ricardo Pietrobon, Guilherme Roberto Zammar, Jatin Shah, Bryan Yoo, Mauro Maldonato, Steven Takemoto, Thomas P. Vail

**Affiliations:** 1 Research on Research Group, Department of Surgery, Duke University, Durham, North Carolina, United States of America; 2 Department of Surgery, Duke University Medical Center, Durham, North Carolina, United States of America; 3 Pontifícia Universidade Católica do Paraná, Curitiba - Paraná, Brazil; 4 Department of Orthopaedic Surgery, University of California San Francisco, San Francisco, California, United States of America; 5 Philip R. Lee Institute for Health Policy Studies, San Francisco, California, United States of America; 6 Stanford University, Palo Alto, California, United States of America; 7 Department of Storic, Linguistic and Antropologic Sciences, University of Basilicata, Potenza, Italy; Sapienza University of Rome, Italy

## Abstract

**Background:**

The Hospital Acquired Condition Strategy (HACS) denies payment for venous thromboembolism (VTE) after total knee arthroplasty (TKA). The intention is to reduce complications and associated costs, while improving the quality of care by mandating VTE prophylaxis. We applied a system dynamics model to estimate the impact of HACS on VTE rates, and potential unintended consequences such as increased rates of bleeding and infection and decreased access for patients who might benefit from TKA.

**Methods and Findings:**

The system dynamics model uses a series of patient stocks including the number needing TKA, deemed ineligible, receiving TKA, and harmed due to surgical complication. The flow of patients between stocks is determined by a series of causal elements such as rates of exclusion, surgery and complications. The number of patients harmed due to VTE, bleeding or exclusion were modeled by year by comparing patient stocks that results in scenarios with and without HACS. The percentage of TKA patients experiencing VTE decreased approximately 3-fold with HACS. This decrease in VTE was offset by an increased rate of bleeding and infection. Moreover, results from the model suggest HACS could exclude 1.5% or half a million patients who might benefit from knee replacement through 2020.

**Conclusion:**

System dynamics modeling indicates HACS will have the intended consequence of reducing VTE rates. However, an unintended consequence of the policy might be increased potential harm resulting from over administration of prophylaxis, as well as exclusion of a large population of patients who might benefit from TKA.

## Introduction

Recent public debate on health care reform has renewed focus on patient safety and cost containment [1]. A value-based health care strategy strives to ‘bend the healthcare cost curve’ by publically reporting rates of post-operative complications and adherence to quality of care and patient safety measures [2]. Value-based systems assume institutions will minimize complication rates thereby costs will be reduced while improving the quality of care. Value-based mandates, however, may have unintended consequences on overall complications, unanticipated costs, or reduced access for individuals who may benefit from a treatment.

As a part of the Deficit Reduction Act of 2005, the Secretary of Health and Human Services (USA) identified high cost and high volume procedures where preventable hospital acquired conditions were reimbursed at higher rates [3]. For example, total knee arthroplasty (TKA) patients who develop venous thromboembolism (VTE), a term used to collectively describe deep vein thrombosis (DVT) and pulmonary embolism (PE), accrue higher costs than those who do not experience this condition. Evidence-based guidelines suggest administering prophylaxis medications (anti-clotting blood thinners) reduces VTE rates [4], [5], [6]. To encourage adherence to these guidelines, beginning on October 1, 2008, the “hospital acquired condition” strategy (HACS) no longer reimburses inpatient costs after a newly diagnosed VTE if recommended prophylaxis was not administered prior to TKA procedures [7].

Every year in the U.S., over 185,000 VTE cases are diagnosed in patients aged 45 years or older [8]. More than 35% occur after surgical events [9], frequently leading to long-term disability or death [10], [11]. Certain clinical conditions, such as malignant neoplasm, trauma, congestive heart failure, obesity, multiparity, advancing age and hypercoagulability increase VTE risk [12], [13], [14]. Despite evidence suggesting its efficacy to reduce complication risk, 41 to 84% of the patients do not receive recommended VTE prophylaxis [15], [16]. Patients receiving TKA have increased risk of VTE, and recommended prophylaxis reduces this risk [17]. Therefore, a policy that encourages anti-thrombotic prophylaxis seems reasonable.

System Dynamics [18] has been used to model the consequences of national smoking cessation initiatives [19], smallpox vaccination [20], and prevention vs. management of chronic disease, [21]. Changes in health care policy can affect access, reimbursement and clinical outcomes. Complex interrelationships have multiple clinical ramifications on patients, payors, providers, and hospital administrators. System Dynamics uses stock and flow diagrams, feedback loops and various ‘what if scenarios’ to portray the casual relationships among these elements and predict long term implications of these complex interactions [22]. For example, improving adherence to prophylaxis guidelines may decrease VTE rates, but this may also increase the rates of bleeding which in turn may increase infection rates. Mandating VTE prophylaxis may reduce access to TKA for patients who have increased risk of bleeding.

This study applies system dynamics to estimate the impact of HACS on rates of VTE and other surgical complications (such as bleeding and infection). We hypothesize that the increased rates of VTE prophylaxis will actually increase rates of bleeding and infection [23]. Additionally, patient subgroups who might benefit from a knee replacement but have increased risk of bleeding will accumulate.

## Methods

### Modeling Context

The Hospital Acquired Condition Strategy (HACS) enacted by federal legislation applied to Medicare reimbursement for VTE [7]. The majority of patients eligible for Medicare are older than age 65, and VTE risk varies considerably by age [24], so patients younger than age 65 were excluded when developing parameters for the model. Typical Medicare reimbursement to hospitals is $10,000, $13,000 for patients with a major comorbidity or those who experience a complication [25]. The rationale for implementing the HACS were the low rates of guideline-recommended proplylaxis [26]. Some argue is neither preventable nor accurately measurable [27]. Nearly half of patients with a DVT detectable by ultrasound do not experience clinical symptoms [28]. Patients with DVT risk factors will more likely receive prophylaxis and can be excluded from HACS policy by indicating “presence on admission can not be determined” [29]. On the other hand, clinicians may be hesitant to administer DVT prophylaxis to patients with increased risk of bleeding so these patients might be affected by the policy change.

### Literature Review

Citations from the Federal Register “hospital acquired condition” policy [7] and the Surgeon General’s Call to Action to prevent DVT and PE [30] were used to define an initial set of risk factors and rates of DVT, PE, and bleeding in various populations as well as potential risk factors. Additional studies were identified through PubMed database by taking the intersection of each risk factor and the terms venous thrombosis, DVT, PE, or DVT prophylaxis.

### Expert Interview

Certain model inputs such as willingness to treat patients with increased risk of DVT or bleeding, rates of surgical site infection in patients with excessive bleeding, and those harmed by DVT and bleeding were not clearly described in the literature. Three arthroplasty surgeons at the University of California, San Francisco (UCSF) were queried to obtain a range for these values through mutual agreement. (Refer Information S1 -Questions asked to expert panel) All three surgeons are board certified knee specialists performing a large volume of total knee replacement procedures in a University hospital setting.

### Model Parameters

**Figure 1:**
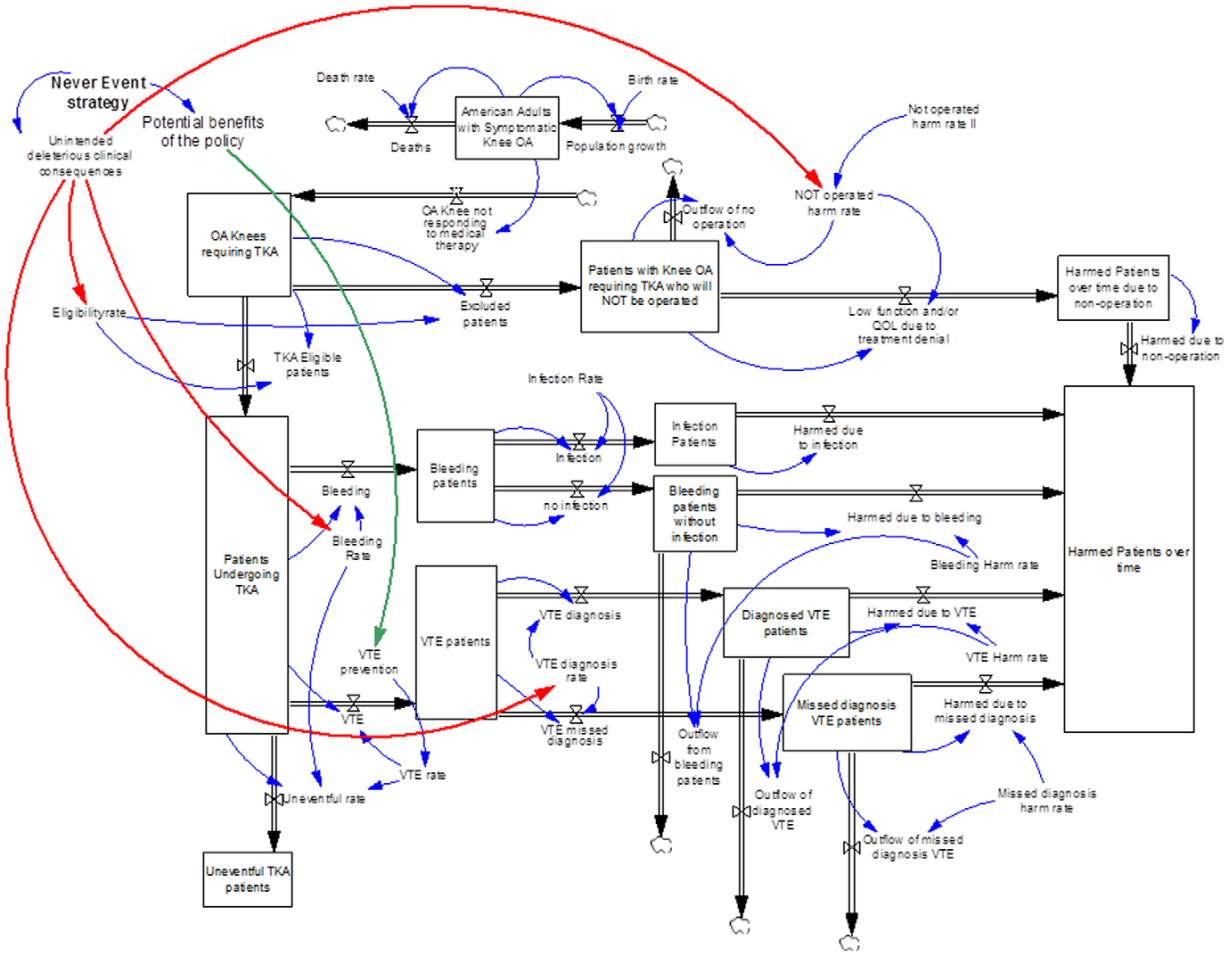
An illustration of the stocks, causal elements, relationships and impact of HACS in the system dynamics model.

The parameters included in the model are either stocks, represented as boxes in Figure 1 or causal elements represented at the ends of arrows. Stocks, accumulate over time with the quantity regulated by influx and efflux rates. For example, the stock of patients who can benefit with TKA increase as the number of patients with osteoarthritis in the overall population increases. Meanwhile, patients deemed ineligible and those who receive TKA decrease this stock. The causal elements affect results derived from the model but do not accumulate over time. An example of a causal element is the percentage of patients receiving TKA or fraction of patients with postoperative bleeding who later developed a surgical site infection.

### Model Relationships

The movement of patients through the system is regulated by the relationships between the stocks and causal elements. A flow is represented by a thick, double-lined arrow emerging out of or entering into a stock. Causal links illustrating the effect of the causal variables are represented by thin arrows. Blue arrows represent causal effects that may increase or decrease flows, red and green arrows indicate the impact of HACS.

### Policy Intervention

We compare two simulation scenarios, with and without HACS. The HACS scenario represents the potential result of withholding reimbursement for hospital care associated with the treatment of VTE when VTE prophylaxis is not administered prior to TKA. VTE rates are those indicated when Enoxaparin, the prophylaxis recommened by both the American College of Chest Physicians (ACCP) [5] and the American Academy of Orthopaedic Surgeons (AAOS) [6], is or is not administered. We assume VTE rates reported in clinical trials would be achieved with increased adherence to VTE prophylaxis guidelines.

### Dynamics Hypothesis

The ideal scenario as envisioned by those who implemented HACS policy is a hypothesis that rates of VTE will decrease over time, while rates of surgical complications and disparity remain stable.

### The Systems Dynamics Model

Vensim software was used to design and simulate the System Dynamics model [18]. The first parameter illustrated in Figure 1 is the stock representing the prevalence (9.7 million) of patients over age 65 suffering with symptomatic knee osteoarthritis (OA) [24]. As the model cycles from 2008 through 2020, the number of patients in this stock is dependent on birth and death rates. The number of patients requiring TKA are those with OA who do not respond to non-operative therapy. These patients could either undergo TKA or be excluded, depending on eligibility requirements. The stock entitled “Patients with Knee OA requiring TKA who will NOT be operated” represents patients with clinical conditions (such as malignant neoplasm, morbid obesity, immobile standing position, hypercoagulable state, etc) who are not eligible for TKA. Patients undergoing TKA could have an uneventful course, a VTE, or a bleeding complication. Patients who experience excess bleeding may develop a surgical site infection resulting from hematoma, evacuation or prolonged wound drainage [31], [32]. Of those experiencing VTE, a fraction will die due to fatal PE, some have to undergo at least 6 months of oral anticoagulation, and those who will not be diagnosed with VTE may experience chronic venous insufficiency requiring long term mechanical compression therapy. We modeled four complication scenarios (bleeding, infection, diagnosed and undiagnosed VTE), with fractions of harmed patients.

The model examined hypothetical simulations over a period of 12 years from 2008 continued through 2020. The stocks examined were the number of patients experiencing VTE, bleeding, infection, or were deemed ineligible for surgery individually, as well as the cumulative number of harmed patients per year. Inputs for the model are indicated in Table 1. The parameters assume a 1.5% decrease (from 14.4–12.9%) in TKA eligibility among patients with OA with HACS. Higher rates of bleeding (9.6% vs. 1.4%) and lower rates of VTE (2 vs. 5%) due to increased administrating VTE prophylaxis with HACS. We also speculate that a fraction of VTE diagnoses (25 vs. 15%) will be missed because of a disincentive with HACS to identify the condition, and that 10% of patients with a missed diagnosis will be harmed. The range of values for each parameter that were modeled in the sensitivity analyses are also indicated in Table 1.

**Table 1:** Model parameters, minimum and maximum range, and reference sources.

	Baseline (%)		Sensitivity Analysis (minimum - maximum value)		Reference
	Without HACS	With HACS	Without HACS	With HACS	
Eligibility rate	14.4%	12.9%	13.4–15.4%	11.9–13.9%	[49], [59], Expert panel
Bleeding rate	1.4%	9.6%	0.4–2.4%	7.6–10.6%	[60]
VTE rate	5%	2%	3–7%	0.5–4%	[21, 61, 62]
VTE diagnosis rate	85%	75%	75–90%	65–85%	Expert panel
Infection rate	10%	10%	5–20%	5–20%	[32], [33], [38] Expert panel,
Bleeding harm rate	58%	58%	46–70%	46–70%	[62]
VTE harm rate	75%	75%	65–85%	65–85%	[63], Expert panel
Missed diagnosis harm rate	10%	10%	5–20%	5–20%	[64], Expert panel

## Results

Model stock outputs indicate HACS results in a 3-fold decrease in VTE rates (Table 2). However, the fraction of HACS with bleeding complications associated is 6-fold higher, and 6-fold more patients are potentially ineligible for TKA per year with HACS in place.

**Table 2:** Model outputs indicating the number of harmed patients per year.

Time (Year)	2008	2011	2014	2017	2020
VTE without HACS	19,500	20,560	21,279	21,679	22,040
VTE with HACS	19,500	8,050	7,661	7,770	7,898
Diagnosed VTE without HACS	16,575	17,151	17,913	18,316	18,631
Diagnosed VTE with HACS	16,575	7,428	5,834	5,803	5,892
Missed VTE without HACS	2,925	3,027	3,161	3,232	3,288
Missed VTE with HACS	2,925	2,359	1,939	1,934	1,964
Bleeding patients without HACS	5,460	5,757	5,958	6,070	6,171
Bleeding patients with HACS	5,460	34,659	36,593	37,288	37,909
Bleeding without infection without HACS	4,914	5,085	5,311	5,430	5,524
Bleeding without infectionwith HACS	4,914	27,238	32,387	33,347	33,931
Infection without HACS	546	565	590	603	614
Infection with HACS	546	3,026	3,602	4,705	3,770
Ineligible patients without HACS	0	0	0	0	0
Ineligible patients with HACS	0	35,751	38,543	39,335	39,993
Total harmed without HACS	0	15,698	17,175	17,689	18,018
Total harmed with HACS	0	51,387	64,038	66,454	67,680

**Figure 2:**
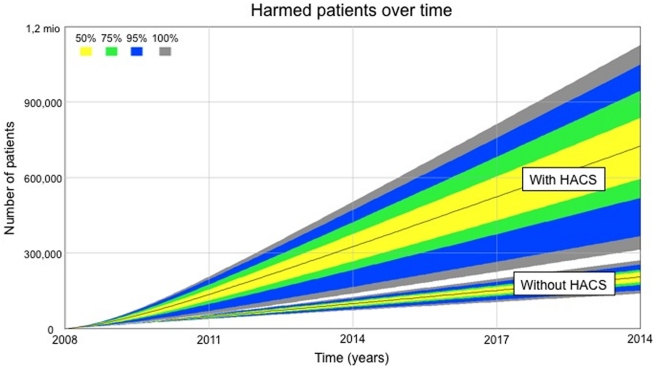
The fraction of patients harmed by HACS over time including sensitivity analysis adjustments.

The cumulative potential harm over time due to VTE, bleeding, infection and denied access that could be attributed to HACS is illustrated in Figure 2. The model indicates the fraction harmed with HACS will be 2.8 times higher in 2011, 3.3 times in 2014, 3.4 times in 2017 and 3.5 times in 2020. The increase in the total number of adults harmed by the HACS reaches half a million people by year 2020. Sensitivity analysis indicates this increase might be as small as 43,000 and as high as 980,000.

**Figure 3:**
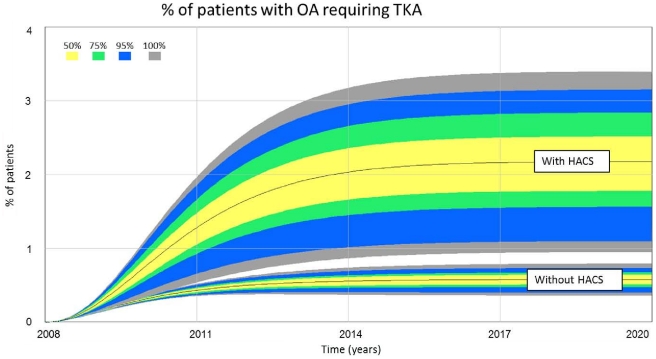
The fraction % of patients with osteoarthritis who might benefit from, but not receive TKA.

The fraction of patients affected by policy stabilizes as the model reaches equilibrium (Figure 3). HACS increased the percentage of patients suffering from OA pain who could benefit from, but do not receive TKA by 1.6% (2.2% with HACS, 0.6% without). Sensitivity analysis indicates the percentage of patient denied TKA with HACS may be as high as 3.4% and as low as 0.95%, and 0.79–0.36% without.

## Discussion

Several interventions [33], including physician alerts [34], decision support informatics [35], and regular audits [36] are shown to increase the rates of VTE prophylaxis. Despite these efforts, only 60% of TKA patients receive enoxaparin as recommended by ACCP guidelines [16]. The monetary incentive mandated by HACS, not reimbursing costs resulting from VTE when VTE prophylaxis was not administered, was effective for decreasing VTE rates, but our model suggests HACS will result in an overall 6-fold increase in complication rates. While others have suggested the possibility of unintended consequences [37], this study indicates half a million people might be harmed by HACS by the year 2020. Furthermore, the fraction of Americans who could benefit, but are denied for TKA is increased 1.6% with HACS because of their risk of developing a VTE complication which would place the providers and hospitals at financial risk for the episode of care.

Mandating VTE prophylaxis increases the risk of prolonged wound drainage, extended hospital stay, and surgical site infection [31], [32], [38]. Surgical site infection measureably reduces health-related quality of life [39]. We did not attempt to estimate whether savings to Medicare by refusing to reimburse care for VTE complications with HACS is offset by the cost of prophylaxis, extended hospitalization and readmission resulting from bleeding and infection complications. Other potential model parameters were not studied as well. The efficacy of recommended prophylaxis to reduce the risk of death due to a PE when compared to aspirin in TKA patients remains controversial [6]. Other prophylaxis regimens may result in a different impact of HACS. Bleeding rates for this study were based on published clinical experience with low molecular weight heparin.

A policy that penalizes the occurrence of adverse outcomes will likely decrease access to at-risk patients. The potential for inequity may be greater than estimated in this model. Kahneman’s Prospect Theory suggests aversion of loss is psychologically twice as powerful as the potential for gain [40]. The desire to avoid HACS consequences could result in overly aggressive VTE prophylaxis, under reporting of VTE, and exclusion of patients who could benefit from TKA. Our model estimates the policy will exclude over 35,000 patients/year. Access to care is driven by perceptions of both the surgeon and patient. Only a third of surveyed patients with painful osteoarthritis were willing to consider TKA as a treatment option [41]. TKA significantly improves the quality of life of patients with osteoarthritis [42], [43], [44]. Typical patients experience a gain of more than one quality adjusted life year (QALY from 6.8 to 8.0 with TKA) [45]. Elderly patients with comorbidity and those living in poverty might be comparable to those who are excluded by HACS. These patients experienced a similar QALY gain, (0.8, 5.8 to 6.6 with TKA) [45].

Either a “carrot” or “stick” approach can be used to provide monetary incentives to improve adherence to recommended care guidelines. “Pay for performance” (P4P) rewards increased adherence to quality metrics [46] while “hospital acquired condition” penalizes undesirable outcomes. The amount of increased guideline adherence varies across studies [47], , and these programs may either exacerbate [50], [51], [52] or reduce racial disparity [46], [49]. Underserved patients may experience significant out-of-pocket costs so they may delay seeking of care, both for the OA leading to TKA, but also for post-surgical monitoring of emerging complications. An alternative to the current approaches for rewarding guideline-based care might be to reward those who provide high quality and equitable access to underserved patients [48], [53].

Although our study quantifies the relative impact of intended and unintended consequences of the HACS policy, the model has some limitations. First, model inputs were based on assumptions drawn from publications. In cases where the data could not be directly extracted from the literature and only approximations were available, expert opinions from three surgeons in an academic healthcare setting were obtained. Surgeons in other settings may have opinions that differ, resulting in a greater or lesser likelihood to treat patients with risk factors. However, the dynamic nature of the model allows changing model parameters whenever desired. Second, the relationship between age and complication rates, or the effectiveness of VTE prophylaxis by risk profile is not well documented in the literature. Consequences of aggressive prophylaxis and the tendency to deny surgery to subgroups of underserved patients were disregarded and would need special attention. Third, we did not stratify complications by severity. So potentially lethal and less harmful complications were included in the same stock.

Our objective was to provide a range of potential outcomes resulting from the Hospital Acquired Condition Strategy. A logical extension could determine how HACS differently impacts various at-risk populations. While it seems logical to propose process variables such as VTE prophylaxis administration for measuring quality of care, it is also clear that VTE is a problematic outcome because it can occur even with proper prophylaxis [54], [55], [56], [57]. Enforcing policies to prevent VTE can decrease access to care and pose a theoretical risk of increasing overall complication rates.

## Supporting Information

Information S1 **Questions asked to expert panel.**
(DOC)
