# Supplementary material for: System Dynamics to Model the Unintended Consequences of Denying Payment for Venous Thromboembolism after Total Knee Arthroplasty
Source: PLoS One. 2012 Apr 20;7(4):e30578. doi: 10.1371/journal.pone.0030578 (PMC3335025; doi:10.1371/journal.pone.0030578)
Supplement: Information S1 — Questions asked to expert panel. (DOC) [file pone.0030578.s001.doc]

# Supporting information

**S1. Questions asked to expert panel**

- What percentage of your patients (range) are excluded because of risk of bleeding?
- What percentage are excluded because of risk of thrombosis?
- What percentage of surgeons in your community would begin non-reporting DVT if it meant their reimbursement would decrease?
- What percentage of bleeding patients develops a surgical site infection due to a postoperative bleeding complication?
- What percentage of your patients are harmed in any way due to a developed DVT?
